# Probiotics in septic acute kidney injury, a double blind, randomized control trial

**DOI:** 10.1080/0886022X.2023.2260003

**Published:** 2023-09-19

**Authors:** Jonathan S. Chávez-Íñiguez, Miguel Ibarra‑Estrada, Alejandro Martínez Gallardo-González, Ari Cisneros-Hernández, Rolando Claure-Del Granado, Gael Chávez-Alonso, Eduardo M. Hernández-Barajas, Alexia C. Romero-Muñoz, Fidel Ramos-Avellaneda, Manuel L. Prieto-Magallanes, Marcela Plascencia-Cruz, Jarumi A. Tanaka-Gutiérrez, Cristina Pérez-Hernández, Guillermo Navarro-Blackaller, Ramón Medina-González, Luz Alcantar-Vallin, Karina Renoirte-López, Guillermo García-García

**Affiliations:** a Nephrology Service, Hospital Civil de Guadalajara Fray Antonio Alcalde, Guadalajara, Jalisco, Mexico; b Health Sciences Center, University of Guadalajara, Guadalajara, Jalisco, Mexico; c Intensive Care Unit, Hospital Civil de Guadalajara Fray Antonio Alcalde, Guadalajara, Jalisco, Mexico; d Division of Nutrition, NIN Institute, Guadalajara, Jalisco, México; e Division of Nephrology, Hospital Obrero #2 – C.N.S, Universidad Mayor de San Simon School of Medicine, Cochabamba, Bolivia

**Keywords:** AKI, sepsis, probiotics, intestinal dysbiosis

## Abstract

**Introduction:**

During acute kidney injury (AKI) due to sepsis, the intestinal microbiota changes to dysbiosis, which affects the kidney function recovery (KFR) and amplifies the injury. Therefore, the administration of probiotics could improve dysbiosis and thereby increase the probability of KFR.

**Methods:**

In this double-blind clinical trial, patients with AKI associated with sepsis were randomized (1:1) to receive probiotics or placebo for 7 consecutive days, with the objectives of evaluate the effect on KFR, mortality, kidney replacement therapy (KRT), urea, urine volume, serum electrolytes and adverse events at day 7.

**Results:**

From February 2019 to March 2022, a total of 92 patients were randomized, 48 to the Probiotic and 44 to Placebo group. When comparing with placebo, those in the Probiotics did not observe a higher KFR (HR 0.93, 0.52–1.68, *p* = 0.81), nor was there a benefit in mortality at 6 months (95% CI 0.32–1.04, *p* = 0.06). With probiotics, urea values decreased significantly, an event not observed with placebo (from 154 to 80 mg/dl, *p* = 0.04 and from 130 to 109 mg/dl, *p* = 0.09, respectively). Urinary volume, need for KRT, electrolyte abnormalities, and adverse events were similar between groups. (ClinicalTrial.gov NCT03877081) (registered 03/15/2019).

**Conclusion:**

In AKI related to sepsis, probiotics for 7 consecutive days did not increase the probability of KFR, nor did other variables related to clinical improvement, although they were safe.

## Introduction

The intestinal microbiota comprises 100 trillion microorganisms, such as bacteria, viruses, fungi, and protozoa, that interact with the human host during health and disease processes [1,2] and have been fundamental in the evolution of humans [3,4]. The great majority of intestinal bacteria (∼90%) are categorized into three groups: Bacteroidetes, Firmicutes, and Actinobacteria. During physiological states, they contribute to the generation of short-chain fatty acids (SCFAs), which considerably interact with the immune system and affect all organ functions. In the kidney, for example, they interact with four SCFA receptors (Gpr41, Gpr43, Gpr109a, and Olfr78) located on different sites of the nephron [5], promoting the physiological functioning of the kidney and maintaining the glomerular filtration rate (GFR) and tubular capacity to reabsorb and secrete solutes, which is a process called symbiosis [6].

During disease and inflammation, the intestinal microbiome undergoes changes in its composition, causing the proliferation of pathogenic bacteria, which in turn promotes more local and systemic inflammation, elevated concentrations of uremic toxins, increased intestinal permeability, endotoxemia, and immunodeficiency [7,8], affecting homeostasis through different pathways. This phenomenon is called intestinal dysbiosis [6] and has been associated with adverse clinical outcomes in experimental models and humans in many different clinical scenarios, such as systemic inflammatory response syndrome [9], sepsis [10], chronic kidney disease (CKD) and acute kidney injury (AKI) [11–14].

AKI occurs in one out of every four hospitalized patients, and 22.8% of these patients die during hospitalization [15,16]. The main cause of hospital-acquired AKI (HA-AKI) is sepsis, which accounts for 70% of cases in our community. Various efforts to identify specific treatments to attenuate sepsis-induced AKI, such as antifibrotics, anti-inflammatory agents and immunomodulators, have been unsuccessful [17,18]; therefore, the management of sepsis-induced AKI is currently limited to treating the main etiology [19] and, in severe cases, correcting its complications through kidney replacement therapies (KRTs) [20]. There is an urgent need to explore alternatives for the treatment of AKI [21].

Since AKI is a syndrome that generates intense systemic inflammation [22], attenuation of this phenomenon has been shown to improve renal function and parenchymal damage [23,24]. AKI and intestinal dysbiosis coexist, amplifying local and systemic inflammation and facilitating the proliferation of harmful intestinal bacteria, which generates a vicious cycle that worsens clinical status and causes kidney injury and subsequent systemic failure [14,25]. It seems reasonable that modulating the microbiota and improving intestinal dysbiosis during AKI by administrating probiotics could improve outcomes in patients with these syndromes. We conducted the first clinical trial of probiotic treatment for patients with sepsis-induced AKI (ClinicalTrial.gov NCT03877081) (registered 03/15/2019) with the hypothesis that by modulating intestinal dysbiosis with probiotics, AKI recovery will improve.

## Methods

### Study participants

This was a single-center double-blind randomized clinical trial that screened all consecutive patients admitted for AKI who met the diagnostic criteria for sepsis and had been evaluated by the Nephrology Department at the Hospital Civil de Guadalajara Fray Antonio Alcalde, a large referral center that cares for patients without health care insurance and with low socioeconomic status in Jalisco, México.

Patients were enrolled from February 2019 to March 2022. Due to the COVID-19 pandemic, we enrolled patients at a slower rate and over a relatively long period (2019 through 2021). The trial was conducted in accordance with the principles of the Declaration of Helsinki and the International Conference on Harmonization Guidelines for Good Clinical Practice. All patients gave their written informed consent before any study-related procedure. The study, which was approved by the local ethics committee (HCG/CEI-1342/18), was prospectively registered in clinicaltrials.gov (NCT03877081) on 03/15/2019. No funding was received to conduct this study.

### Definitions

AKI was defined as an increase in serum creatinine (sCr) levels according to Kidney Disease Improving Global Outcomes (KDIGO) [26], and CKD was defined according to the KDIGO guidelines [27]. The estimated glomerular filtration rate (eGFR) in ml/min/1.73 m^2^ was calculated according to the Chronic Kidney Disease Epidemiology Collaboration (CKD-EPI) equation [28]. Baseline eGFR was considered according to the more recent sCr level measured within the prior 3 months. For patients without baseline sCr values, we estimated it by back-calculating the Modification of Diet in Renal Disease (MDRD) equation, assuming eGFR 100 mL/min/1.73 m; this surrogate was preferred because it has been shown to be more accurate than assuming 75 mL/min/1.73 m^2^ [29]. Sepsis was defined according to the Sepsis-3 criteria [30]. Kidney function recovery (KFR) was defined as the return of sCr levels to <0.30 mg/dL from the baseline value after up to 7 days of follow-up. Seven days was chosen to define renal recovery since after day 7, if patients do not recover from AKI, they are considered to have developed acute kidney disease (AKD), and they have an increased risk of adverse outcomes [31,32].

All comorbidities and clinical data were prospectively collected during the first evaluation.

Adverse events were prespecified according to those most frequently reported with the use of probiotics, such as abdominal distension, nausea, rash, vomiting and diarrhea [33], and they were prospectively recorded on a daily basis by the nephrology staff. Additional data were collected from the medical records and hospital electronic database. Appropriate adherence to treatment was defined as >80% of the administered capsules being consumed.

### Study outcomes

The primary outcome was KFR on day 7 (sCr return to <0.30 mg/dL from the baseline value). Secondary outcomes included variables related to KFR during the treatment and the follow-up period, namely, the change in urinary volume, percentage of decrease in sCr levels, in-hospital mortality, mortality during follow-up, KRT requirement, electrolyte levels and acid–base abnormalities. The prespecified adverse events mentioned above were prospectively reported.

### Randomization and treatment assignments

Randomization was carried out by a computer-generated stratified sequence with a 1:1 allocation ratio in blocks of 5, with the groups stratified by sex. The researchers, who used a concealed opaque envelope system, performed group assignment after informed consent was obtained. A double-blind, double-dummy design was used. The nephrology staff administered the treatment in white bottles that were only marked with the patient’s assignment number.

Given the lack of previous clinical trials on this topic, a formal sample size calculation was not performed, and we chose a convenient sample size of 92 patients. All patients received the personalized management suggested by the KDIGO AKI bundle of care guidelines [26]. The study design is described in Supplemental Figure 1. Inclusion criteria were age >18 years, the presence of sepsis-induced AKI, willingness to participate and signed informed consent. Patients with CKD KDIGO stages 4–5 or on chronic dialysis, kidney transplant, pregnancy, or who had not signed the informed consent form were excluded.

Patients who met the inclusion criteria and signed informed consent were randomized to the intervention group (probiotics) or the control group (placebo), and 2 capsules *per os* (or through an enteral tube) were administered every 24 h. Blood and urine tests were performed to measure the variables of interest every 24 h and were processed in a certified central laboratory.

The results are reported following the CONSORT guidelines for clinical trials.

### Interventions

Patients in the intervention group received 2 capsules of Simbin-RNL® or 2 capsules of placebo (maltodextrin) every 24 h for 7 consecutive days. The gastro-resistant Simbin-RNL® capsule contained 540 mg of a mixture of *Streptococcus thermophilus, Lactobacillus acidophilus, Bifidobacterium longum* (90 billion colony forming units (CFU) in the 2-capsule serving (4.5 x10e10 CFU per capsule), agave inulin (the contribution of prebiotic fiber per serving is 600 mg per 2 capsules), magnesium stearate and silicon dioxide. The Simbin-RNL® formula comprises a mixture of a food supplement of probiotic strains and agave inulin (a prebiotic) that acts as soluble fiber, which arrives intact in the intestine to be used as food for the anaerobic intestinal microbiota, promoting the growth of saccharolytic bacteria and increasing the concentration of SCFAs; it also promotes alterations in intestinal pH, inhibition of pathogens *via* the generation of antibacterial compounds, competitive elimination of pathogens in receiver binding sites and competitive for available nutrients [7]. This formula was chosen for this trial since it has shown kidney function benefits in experimental models of AKI, improving kidney function measured by sCr and urea levels and attenuating histological injury [10,34].

### Statistical analysis

Categorical variables are presented as numbers and percentages, and comparisons between groups were performed with the chi-square or Fisher exact test as appropriate. The Shapiro–Wilk test was performed to assess data distribution; continuous variables are summarized as the means ± standard deviations (SD) if the data were normally distributed or medians and interquartile ranges (25–75th) if the data were nonnormally distributed and were compared using Student’s t test or the Mann–Whitney U test, respectively. For variables measured at multiple time points, repeated measures analysis of variance tests were used for comparisons between groups. Time to renal recovery and time to death were both plotted on Kaplan–Meier curves, and the groups were compared with the log-rank test. A multiple regression analysis was performed with the enter method, and all the baseline variables with a p value ≤0.20 were entered into the model in bivariate analysis. All tests were two-tailed, and results with a p value less than 0.05 were considered significant. Statistical analysis was performed, and graphics were generated with MedCalc Statistical Software (Ostend, Belgium. Ver 19.1.3) and GraphPad Prism (California, USA. Ver 9.2.0), respectively.

## Results

**Figure 1.**
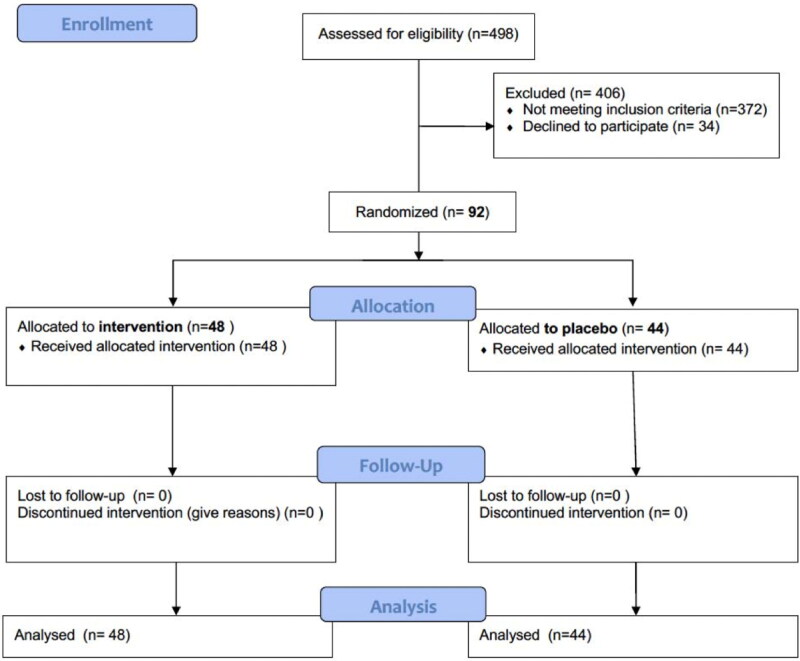
CONSORT Flow diagram.

From February 2019 to March 2022, 621 patients with AKI underwent nephrology consultation, and 123 did not have sepsis or they lacked variables of interest for the analysis; thus, 498 were assessed for eligibility, among whom 372 did not meet inclusion criteria, and 34 did not sign the consent form; therefore, 92 patients were randomized, with 48 in the intervention group (probiotics) and 44 in the placebo group (Figure 1).

The baseline demographic characteristics of the randomized study participants are described in Table 1. The mean age was 56.9 ± 16.3 years, 51% [35] were female, almost half of them had diabetes (46%), and a quarter had CKD (27%). We did not observe any severe electrolyte alterations, and all had mild metabolic acidosis (pH and HCO_3_, 7.33 ± 0.06 and 19.1 ± 3.9, respectively). Most of the patients (92%) had severe AKI (KDIGO stages 2 and 3, 15% and 77%, respectively) with a mean Sequential Organ Failure Assessment (SOFA) score of 6 [4–8], and 9.3% had septic shock.

**Table 1.** Study baseline characteristics according to the probiotics or placebo groups.

	All (*n* = 92)	Probiotics (*n* = 48)	Control (*n* = 44)	*P*
Age (years)—mean (SD)	56.9 ± 16.3	55.5 ± 16.7	58.4 ± 15.9	0.39
Female (%)	47 (51)	25 (52)	22 (50)	0.84
Comorbidities				
Diabetes (%)	42 (46)	27 (56)	23 (52)	0.70
Hypertension (%)	38 (41)	20 (42)	18 (41)	0.94
Chronic kidney disease (%)	25 (27)	11 (23)	14 (32)	0.34
Chronic heart failure (%)	5 (5)	2 (4)	3 (7)	0.57
Cancer/neoplasia (%)	6 (6)	3 (6)	3 (7)	0.62
Hematological malignancy (%)	2 (2)	1 (2)	1 (2)	0.88
Gastrointestinal disease (%)	12 (13)	6 (12)	(16)	0.74
Neurological disease (%)	9 (6)	4 (8)	5 (11)	0.40
Baseline sCr (mg/dL)	1.1 (0.8–1.6)	1.1 (0.8–1.6)	1.1 (0.8–1.8)	0.82
Day 1				
sCr, mg/dL—mean (IQR)	3.4 (2.3–5.0)	3.7 (2.3-–5.5)	2.9 (2.2–4.5)	0.26
Urea, mg/dL—mean (IQR)	137 (81–187)	154 (83–189)	130 (70–173)	0.26
Urinary volume, ml/day—mean (IQR)	1090 (500–1470)	1000 (500–1500)	1095 (600–1400)	0.87
Sodium, mEq/L—mean (SD)	135 ± 8.6	133 ± 9	136 ± 8	0.09
Potassium, mEq/L—mean (IQR)	4.4 (3.7–5.1)	4.5 (3.9–5.2)	4.1 (3.6–4.7)	0.19
Chloride, mEq/L—mean (SD)	102 ± 9.2	101 ± 9.4	103 ± 9.1	0.37
Calcium, mg/dL—mean (IQR)	7.8 (7.1–8.3)	7.7 (7.0–8.1)	7.9 (7.4–8.8)	0.22
pH—mean (DE)	7.33 ± 0.06	7.33 ± 0.06	7.33 ± 0.07	0.81
Bicarbonate, mmol/L—mean (SD)	19.1 ± 3.9	18.8 ± 3.9	19.6 ± 3.8	0.49
PCO_2_, mmHg—mean (IQR)	35 (29–40)	33 (30–40)	36 (29–44)	0.53
Lactate, mmol/L—mean (IQR)	1.2 (0.9–1.6)	1.3 (0.9–1.6)	1.1 (1.0–1.4)	0.94
Hemoglobin, g/dL—mean (RIQ)	10.2 (8.6–12.0)	10.2 (8.4–11.7)	10.2 (8.9–12.5)	0.62
Leucocytes, 10^3^cél/µL—mean (IQR)	13.2 (9.3–17.9)	13.4 (9.9–18.4)	12.6 (9.1–17.6)	0.52
Platelets, 10^3^cél/µL—mean (IQR)	212 (132–305)	212 (130–321)	194 (136–289)	0.82
Systolic blood pressure (mmHg)	110 ± 19	111 ± 19	109 ± 20	0.63
Diastolic blood pressure (mmHg)	65 (60–79)	60 (60–74)	70 (60–80)	0.16
Cardiac frequency (min)	85 (80–98)	90 (80–100)	82 (78–92)	0.19
Respiratory frequency (min)	20 (18–21)	20 (19–21)	20 (18–21)	0.60
KDIGO n,(%)				
1	7 (7.6)	1 (2.1)	6 (13.6)	0.05
2	14 (15)	7 (14)	7 (16)	1.0
3	71 (77)	40 (83)	31 (70)	0.14
SOFA—mean (IQR)	6 (4–8)	6 (5–8)	5 (4–7)	0.43
Procalcitonin—mean (IQR)	9.3 (2.4–37.4)	8.1 (2.1–34.5)	12.7 (2.6–46.0)	0.76
Septic shock (%)	9.3 (2.4–37.4)	18 (37)	16 (36)	0.91

Regarding adherence, 81% in the probiotic group and 77% in the control group consumed at least 80% of the doses (*p* = 0.63).

### Primary outcome

**Figure 2.**
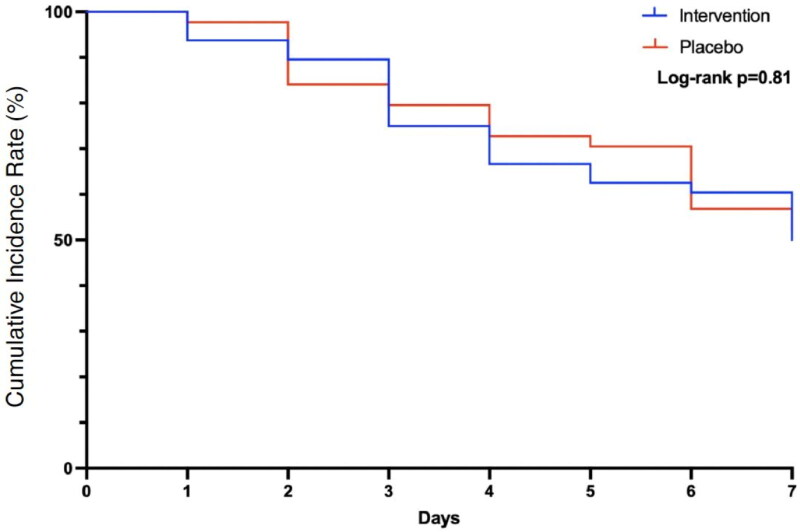
Primary objective, kidney function recovery during the 7 days of the study trial.

The KFR by day 7 is presented in Table 2 and Figure 2. A total of 40 (43%) patients had KFR, including 25 (50%) in the probiotic group and 21 (48%) in the control group, and there was no significant difference (*p* = 0.82) between groups. The relative risk for recovery on day 7 in the intervention group was 0.93 (95% CI 0.52–1.68, *p* = 0.81). Thus, no benefit was observed in patients who received probiotics in terms of improvements in kidney function after an episode of AKI.

**Table 2.** Primary and Secondary objectives.

	All (*n* = 92)	Probiotics (*n* = 48)	Control (*n* = 44)	*p*	RR
Primary Objective					
Kidney function recovery—n (%)*	40 (43)	24 (50)	21 (48)	0.82	1.04
Secondary objective					
Dead – *n* (%)	45 (49)	18 (37)	27 (61)	0.02	0.61
Kidney replacement therapy – *n* (%)	17 (19)	12 (26)	5 (12)	0.11	2.19
Urea, mg/dL –mean (RIQ)	108 (148–232)	80 (31–148)	109 (53–160)	0.40	
sCr – mean (IQR)	2.0 (0.8–2.8)	1.7 (0.7–3.2)	2.2 (1.2–2.7)	0.31	
Urinary volume, ml/día – mean (IQR)	1200 (900–1725)	1125 (750–1400)	1750 (1204–2375)	0.01	
Potassium, mEq/L – mean (IQR)	4.0 (3.5–4.5)	4.0 (3.6–5.0)	4.0 (3.5–4.5)	0.52	
Sodium, mEq/L – mean (SD)	136 ± 6.1	134 ± 5.9	137 ± 5.8	0.01	
Chloride, mEq/L – mean (SD)	102 ± 7.8	101 ± 7.6	104 ± 7.7	0.16	
pH – media (SD)	7.38 ± 0.11	7.35 ± 0.13	7.40 ± 0.07	0.18	
Bicarbonate, mmol/L – mean (SD)	22.2 ± 4.7	21.6 ± 4.6	22.9 ± 4.9	0.45	
Follow-up, days – mean (IQR)	382 (193–967)	642 (227–986)	370 (150–822)	0.20	
Last eGFR/ml/min 1.73m2 – mean CKD-EPI (IQR)	39 (23–94)	46 (23–99)	33 (24–59)	0.45	

### Secondary outcomes

**Figure 3.**
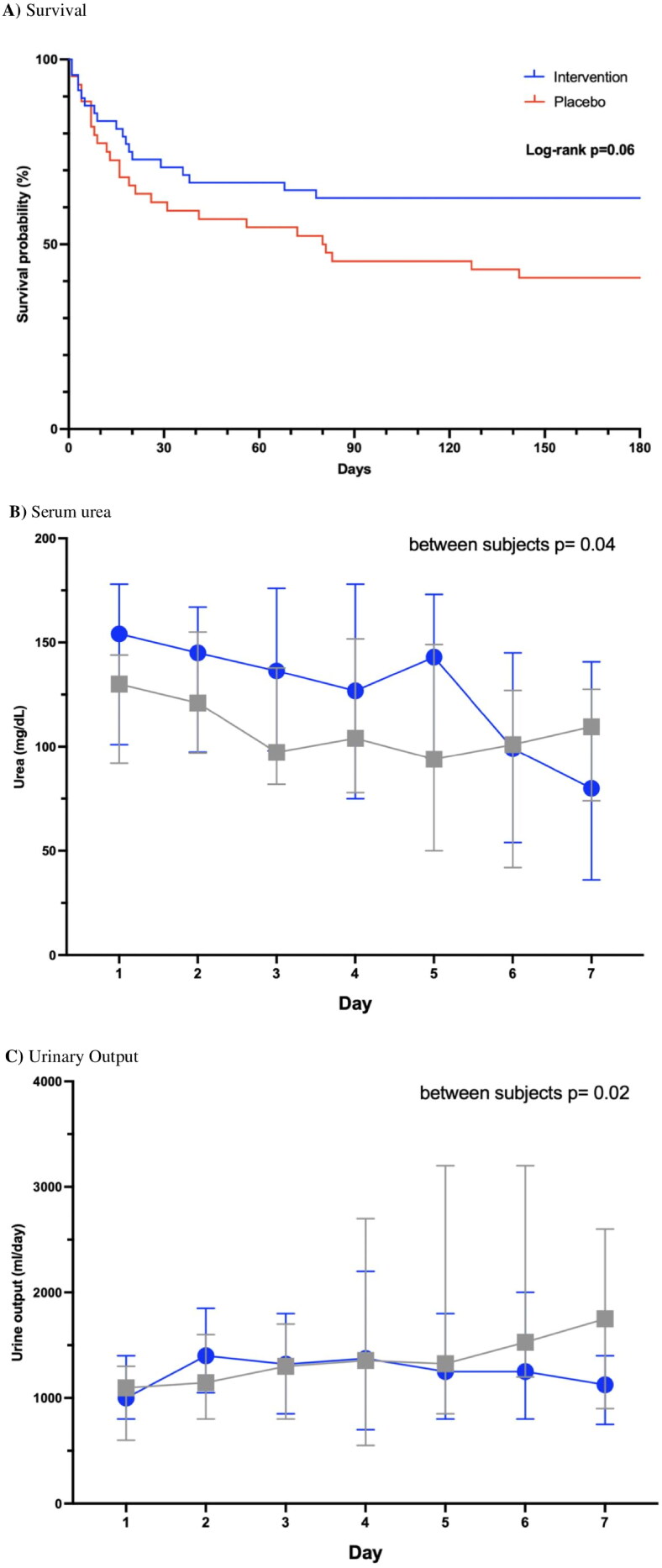
Secondary outcomes, A) Survival, B) serum urea, and C) urinary output, during the 7 days of the study trial.

The results of the secondary outcomes are shown in Table 2 and Figure 3. Among all patients, 45 (49%) died during the study, including 18 (37%) in the probiotic group and 27 (61%) in the placebo group, which indicated that probiotic treatment was favorable with a relative risk (RR) of 0.61 (95% CI 0.39–0.94, *p* = 0.02). Kaplan–Meier survival analysis on day 180 showed a hazard ratio (HR) of 0.56 (95% CI 0.32–1.04 *p* = 0.06) (Figure 3A). Causes of death were similar between the groups, with sepsis being the most common (39%), followed by cancer/neoplasia (13%) or cardiorespiratory (11%), hematological (9%), gastrointestinal (9%) and neurological (6%) disease.

A total of 17 (19%) patients required KRT during the study follow-up period, which was mostly due to uremia, volume overload, and electrolyte abnormalities; this included 12 (26%) in the probiotic group and 5 (12) in the placebo group, with an RR of 2.19 (95% CI 0.84–5.72, *p* = 0.11), as shown in Table 2.

Additionally, urea levels (mg/dL) decreased significantly in the probiotic group from 154 (70–173) to 80 (31–148), which was not observed in the placebo group, in which they only decreased from 130 (70–173) to 109 (53–160), (*p* = 0.09), confirming that the decrease was greater with probiotics (between subjects p value = 0.04) (Table 2, Figure 3B).

Urinary volume (ml/day) increased from 1,000 (500–1,500) to 1,100 (750–1,400) in the intervention group (*p* = 0.65) and from 1,095 (600–1,400) to 1,750 (1,200–2,300) in the placebo group (*p* = 0.05), with a significant difference between the groups (Table 2, Figure 3C).

The patients were followed for a median of 382 days (193–967), and it was observed that their renal function deteriorated, which was based on an overall eGFR of 39 (23–94 mL/min/1.73 m^2^). The eGFR was 46 (23–99 mL/min/1.73 m^2^) in the probiotic group and 33 (24–59 mL/min/1.^73 m2^) in the control group, meeting the criteria for CKD grade 3a and 3b, respectively, without a significant difference between groups (Table 2).

In an exploratory multiple regression analysis, including baseline sodium and potassium levels, heart rate, diastolic blood pressure and stages KDIGO 3 in the model and weighting by group, the results for renal recovery and mortality remained nonsignificant, with *p* = 0.51 and 0.19, respectively.

### Additional outcomes of interest

Potassium levels decreased, and chloride levels increased during the study in both groups, but they did not differ significantly between intervention groups. Only sodium levels decreased in the probiotic group (134 ± 5.9), and they increased in the placebo group (137 ± 5.8) (*p* = 0.01). Calcium values remained stable during the intervention, with no differences between groups (Table 2, Supplemental Figure 2). Acid–base status, which was assessed by serum pH and bicarbonate levels, improved with increasing pH and bicarbonate during the study, with no difference between groups (Table 2, Supplemental Figure 2).

### Adverse events

The prespecified adverse events during the study period are presented in Table 3. A total of 53 patients were documented, and gastrointestinal symptoms predominated. All were considered mild, and they were similar between both groups of the study; none warranted suspending the interventions. The probiotic group presented 34 adverse events; of these, 7 patients presented >2 events, and 27 presented only one event. Abdominal distention was the most common, with 8 reported cases, followed by nausea and diarrhea, with 6 cases each. In the placebo group, 31 presented an adverse event, 6 patients had >2, and the most common was diarrhea with 9 reported cases, there 7 cases of abdominal distention, and 6 cases of vomiting.

**Table 3.** Adverse events during the 7 days of study period.

	Probiotics (34)	Placebo (31)
Diarrhea	6	9
Abdominal distention	8	7
Nausea	7	5
Vomiting	5	6
Rash	3	0
other	5	4

## Discussion

In this double-blind randomized clinical trial carried out in patients with AKI secondary to sepsis, we found for the first time that the administration of probiotics for 7 days did not improve KFR compared with placebo treatment, but it had a trend to decrease the mortality rate, in addition to having an acceptable safety profile.

KFR was observed in half of the patients in this study during the 7-day period, which was similar to what was previously reported for patients with sepsis-induced AKI [36,37]. In this clinical scenario, it is important to implement measures focused on improving kidney recovery within 7 days; if this is not done, there is an increased risk of progression to AKD, which increases the risk of developing *de novo* CKD or CKD progression and increases the risk of cardiovascular complications and death [37]. Recovery of kidney function has been an unresolved issue for many years and has been recognized as a priority [38]; thus far, there is no available treatment that has consistently achieved this objective.

It is plausible to think that uremic toxins derived from intestinal dysbiosis promote kidney dysfunction and fibrosis, a clear example is Trimethylamine-N-oxide (TMAO), a gut microbial-dependent metabolite, plays a direct contributory role in the development and progression of CKD and was even associated with an increased risk of dying [39]. In this study, the lack of efficacy of probiotics in promoting KFR is difficult to contrast with other results since there have been no other similar trials. However, previous experimental models have been encouraging. In mice induced to exhibit pyelonephritis with *E. coli* injection, it was shown that the administration of *Lactobacillus acidophilus* and *Bifidobacterium* before and after sepsis significantly improved renal function and attenuated inflammation and renal fibrosis [40]. Similarly, administration of SCFAs improved renal function after AKI, which was an effect mediated by the decrease in sCr and urea levels, and it also improved the percentage of necrosis seen in kidney biopsies. These improvements were associated with an attenuation of inflammation and significantly lower levels of IL-1b, IL-6, TNFα and MCP-1 [41]. The administration of *Lactobacillus salivarius* following cisplatin-induced AKI decreased the levels of markers of inflammation and severity scores on kidney histology [42] and, interestingly, maintained intestinal wall permeability, suggesting that it would prevent bacterial translocation to the portal circulation and thereby modulate systemic inflammation [43]. SCFA involvement has also been implicated in AKI in humans; it was observed that after AKI, the levels of D-amino acids and especially D-serine increase, which are produced from SCFAs, suggesting a physiological mechanism of protection against kidney insult [44].

We showed that the administration of probiotics tended to decrease mortality in rats. AKI related to sepsis has a poor prognosis. A meta-analysis reported that 45% of affected patients die during their stay in intensive care units and up to 49% die during hospitalization [45]; thus, it is extremely important to try to reduce these numbers. Probiotics have also been shown to decrease mortality in experimental models. In rats with abdominal sepsis induced by cecal ligation, it was shown that the administration of the probiotics *Lactobacillus rhamnosus* and *Bifidobacterium longum* for 7 days decreased the risk of dying by 40% [46]. The involvement of intestinal dysbiosis and colon-associated uremic toxin production in AKI patients was related to AKI severity and an increased likelihood of dying [11]. Even in patients with hospital-acquired AKI, it has been seen that the highest concentrations of uremic toxins generated from intestinal dysbiosis, such as indoxyl sulfate, were associated with an almost 3-fold increase in the risk of dying [47], and a reduction in their levels improved AKI, as evaluated by the Risk, Injury, and Failure; and Loss; and End-stage kidney disease (RIFLE) classification [35]. Our results indicate that a significant difference in mortality might be observable with an increase in the number of cases and an extension of the observation period. In other words, there is insufficient evidence to conclude that the administration of probiotics is not effective at this point.

Regarding other parameters that have been used to evaluate renal function in patients with AKI, such as the need for KRT and sCr concentrations, we did not observe a positive effect of the administration of probiotics. However, serum urea concentrations only improved significantly in the probiotic group, not in the placebo group, although no significant differences were found between them. This effect could be explained by the modulation of intestinal dysbiosis with probiotics and thus the attenuation of urea generation by intestinal bacteria [8], especially in the context of AKI associated with sepsis [48]. Uremia and other colon-derived toxins have an impact on the KFR [35] and mortality [47]. The decrease in urea levels in AKI has been the subject of debate for decades, but recent clinical trials have considered urea levels >240 mg/dL for the decision of when to start KRT in AKI patients (ELAIN and AKIKI2 trials) [49,50]; thus, a decrease in urea levels could be relevant by delaying the start of KRT in certain scenarios.

The finding of higher urinary volume and serum sodium levels in the placebo group than in the probiotics group could be explained by the excretion of free water and thus vascular decongestion. We believe this difference does not profoundly impact the clinical course of these patients since urinary volume and sodium remained within ranges considered safe [51,52].

It is important to comment on the values of eGFR observed during the long-term follow-up of these patients (∼1 year), which was 39 mL/min/1.73 m^2^, with no differences between the study groups, which means that they would be classified as having CKD G3a, which implies a deterioration to almost half their baseline eGFR, which was ∼74 mL/min/1.73 m^2^. The devastating sequelae in renal function after an episode of sepsis-induced AKI have been previously demonstrated and have one of the worst adverse long-term prognoses [53–55].

Considering the reported adverse events, we believe that the administration of probiotics to patients with sepsis-induced AKI was well tolerated and had an acceptable safety level. No adverse events were considered serious, and none of the patients stopped treatment due to any adverse events reported.

For decades, different therapeutic agents have been investigated for the management of AKI associated with sepsis with disappointing results; the use of agents such as statins [55], erythropoietin [56], steroids [57], alkaline phosphatase [58] and pirfenidone [17] is an important justified effort, and the search for a drug that consistently improves kidney function and potentially decreases the probability of dying continues.

## Limitations and strengths

Our results must be interpreted with caution, as this was a single-center study without an *a priori* calculation of sample size due to the lack of literature to estimate an expected minimal clinically important difference between groups; thus, a type II error cannot be ruled out; for instance, according to the observed difference in the primary outcome between groups, the *post hoc* calculated power was 50% in our sample, maintaining an α-error probability of 5%. There was also a lack of measurements of the intestinal microbiota in feces, as well as systemic inflammation parameters, and biomarkers of renal tubular damage that reflect true kidney injury were not assessed. All patients were receiving antibiotics, which may have impacted the probiotics administered. The study population has concomitant pathologies such as CKD, DM, sepsis, and a complex internal environment that could have limited the effect of probiotics. We do not confirm that the absorption of probiotics has been optimal.

The strengths of the study lie in its design and the adequate adherence of the patient groups to treatment; to our knowledge, this is the first randomized control trial of AKI septic patients treated with probiotics [10,34].

In the next study regarding this topic, we suggest measuring the microbiome between groups to quantify an actual difference between those administered the probiotics vs placebo, measure inflammatory markers given the speculation around inflammation and immune regulation. Lastly, a larger and multicenter trial.

## Conclusion

In AKI associated with sepsis, the administration of probiotics for 7 days was safe, and compared with placebo, it did not improve renal function, but there was a trend toward decreased mortality.

## Data Availability

Are available in the historical archive of the Hospital Civil Fray Antonio Alcalde. If any information is requested, please contact Principal Investigator Dr. Jonathan Chávez-Iñiguez (jonarchi_10@hotmail.com).
